# Field Trial of Factors Associated With the Presence of Dead and Non-ambulatory Pigs During Transport Across Three Colombian Slaughterhouses

**DOI:** 10.3389/fvets.2022.790570

**Published:** 2022-01-24

**Authors:** Marlyn H. Romero, Jorge Alberto Sánchez, Rick Obrian Hernandez

**Affiliations:** ^1^Department of Animal Health, Faculty of Agrarian and Animal Sciences, University of Caldas, Manizales, Colombia; ^2^Faculty of Agrarian and Animal Sciences, University of Caldas, Manizales, Colombia

**Keywords:** preslaughter, pigs, transport losses, physiological stress, animal welfare

## Abstract

Transport by land is an essential component for the commercialization of fattening pigs and can have a negative impact on animal welfare. In slaughterhouses, the presence of dead and non-ambulatory animals is an indicator of poor welfare during transport. The objective of the study was to identify risk factors associated with the frequency of dead and non-ambulatory pigs during transport. A survey was conducted in three Colombian slaughterhouses. Data were collected from 372 batches (*n* = 18,437 gilts barrows) and transported directly from the farms to the slaughterhouses. Each truck was individually evaluated; a structured survey was administered to drivers, non-ambulatory and dead pigs on arrival were identified and blood samples were obtained from non-ambulatory pigs to assess physiological indicators of stress. Mortality rates per batch at arrival ranged from 0.08 to 0.17% and prevalence of non-ambulatory pigs per batch ranged from 0.84 to 1.37%.The results of the multilevel mixed effects linear regression model identified the following as risk factors associated with the frequency of total transport losses: truck speed (*P* = 0.04), distance (*P* < 0.01), transport time (*P* < 0.01), load size (*P* < 0.01) and the driver (*P* < 0.01) including the farm as a fixed effect. This study identified risk factors that increased the probability of total transport losses during land transport under Colombian commercial conditions. But more research that involves commercial drivers is needed to develop effective strategies to improve Colombian pig's transportation chain.

## Introduction

Land transport represents a multifactorial challenge for animals and is considered as an stressful time for pigs due to factors associated with food and water deprivation, environmental changes, social mixing, noise, contact with unknown handlers and the truck's microclimate, among others (1). During transport, animals have to make physiological and behavioral adaptations to respond to these stress factors (2). Stress during transport can affect the physiological condition of the animal, as well as its behavior (3). European legislation (4), state that “all animals shall be transported in conditions guaranteed not to cause them injury”, however, animals sent for slaughter with pre-existing conditions (umbilical hernia, non-stabilized fracture, bone fractures, lameness, arthritic joint, respiratory pathology, mastitis, metritis, enteritis, weakness, and others) are more likely to die in transit, become non-ambulatory, or be euthanized on arrival than those that are healthy (5, 6). A study in Canada have shown that sometimes, animals that suffer from certain conditions that compromise their ability to cope with transport (animals with painful conditions), can be transported if certain measures are taken, e.g., if sent for slaughter, they can only be transported locally and directly to the nearest suitable slaughterhouse, also, they must be segregated, loaded last, and unloaded first (7). However, mitigation measures to avoid additional suffering are debatable, at least until there is a consensus over the definition of the term “suffering” (8–10). Therefore, further research would be recommendable to provide new scientific knowledge on the subject, especially when applied to different handling conditions.

Grandin (11) defined compromised pigs as animals with reduced capacity to withstand handling or transport. Categories of compromised pigs observed in slaughterhouses after transport, are the so-called non-ambulatory non-injured (NANI) pigs and non-ambulatory injured pigs (NAI) (6). The incidence of NANI, NAI and dead pigs on-arrival (DOAs) at the slaughterhouses can be a simple indicator to evaluate and identify on-farm pig management problems, as well as problems in preparation for transport (12), loading (2) and transport conditions (travel time, space allowance and within truck climate control (13). For producers, although these conditions have a low incidence, they represent important economic losses related to mortality, total or partial seizure of the carcass, as well as handling and final disposal costs (14).

As reviewed by Grandin (2016) (15), the fitness of animals for transport has no legal definition, which is a challenge for the development and validation of direct indicators for auditing and measurement. Nevertheless, it is a condition that should be systematically assessed in the pre-slaughter logistics chain because there is a risk that animals' pre-existing conditions will be aggravated during the journey and that slaughter will cause suffering (6). However, current guidelines and regulations do not always ensure that only fit animals are transported (7). Although there has been a lot of research on animal welfare during the commercial transport of fattening pigs (2, 4, 7); the study of risk factors associated with the ability of animals to respond to challenges that may occur during transport and slaughter is relevant to identify weaknesses and vulnerabilities that allow the industry to improve its practices in order to make animal handling more efficient, and for government entities to obtain information to validate and adjust current sanitary legislation (11). In Colombia (16) and other Latin American countries, several initiatives, including research and development, increased stakeholder awareness and implementation of legislation and recommendations, have been carried out to promote animal welfare and meat quality (17). However, it is clear that to promote long-term progress in this field it is important to deliver practical solutions, ensuring that they are tailored to the conditions specific to these countries. The objective of the present study was to identify risk factors associated with the presence of dead and non-ambulatory pigs under Colombian pre-slaughter conditions.

## Materials and Methods

The study was carried out in three commercial slaughterhouses in Colombian Central Andes from May to July 2017. The slaughterhouses complied with the announcement 1,500 that created the Official System of Meat Inspection, Surveillance and Control for all meat and meat products and established the sanitary and safety requirements for primary production, slaughtering, processing, storage, transport, sales, import and export of all meat and meat products (18). The animals were transported and slaughtered in compliance with national regulations applied in research and commercial slaughtering (18). The permission to conduct the study was approved by the Ethics Committee for Animal Experimentation of the University of Caldas (Act 1 14/07/2015, -Activities with minimal risk-).

### Study Description

Data was collected from 372 batches (*n* = 18,437 gilts and barrows from commercial lineages) that were transported directly from farms (*n* = 151) to three commercial slaughterhouses: A (*n* = 141 batches; 7,933 pigs, BW=134. 4 ± 0.91 kg), B (*n* = 168 batches; 5,910 pigs, BW=107.4 ± 1.42 kg) and C (*n* = 63 batches; 4,594 pigs, BW=113.6 ± 0.82 kg), respectively. Table 1 presents the characteristics of the three slaughterhouses evaluated in this study. The evaluated pigs were taken from 151 farms located in the departments of Antioquia, Caldas and Risaralda (17 municipalities) in SA, Cundinamarca, Caldas, Meta, Boyacá y Valle (57 municipalities) in SB, and Caldas, Risaralda and Quindío (7 municipalities) in SC. Temperature data in all slaughterhouses was obtained by information from National climatic stations in the proximity of each slaughterhouse. Pre-sorting before loading was done in all farms (Table 2).

**Table 1:** Characteristics of the three slaughterhouses evaluated in this study.

**Variables**	**SA**	**SB**	**SC**
Location	Antioquia (6°13′00″N 75°34′00″W)	Bogotá (4°35′56″N 74°04′51″W)	Caldas (5°06′N; 75°33′O)
Farm System	Main producer of fattening pigs (64.6%)	9.1%	2.6%
Altitude (m.a.s.l)	2,550	2,630	2,038
Mean annual rainfall (mm/year)	2,060	840	1,878
Mean Temperature (°C)	16.6	14.0	15.9
Max (°C)	19.1	16.3	18.5
Min (°C)	11.4	10.9	12.8
**Ventilation system of surveyed trucks**			
Active % (*n*)	0	2.4% (4)	0
Passive % (*n*)	100% (141)	97.6% (164)	100% (63)
Slaughter capacity (pigs/day)	1,000	1,500	350
Slaughter rate (pigs/h)	120	150	35
Unloading ramps	Concrete non-slip floors	Concrete non-slip floors	Adjustable-slope metal ramp (8 m length) with an anti-skid floor
Driving tools	Flags	Electrical prod	Electrical prod
Stunning technique	Head-to-chest electrical stunner (model 11001.1, Sulmaq) in a 1.52 x 0.57 x 1 m box	CO_2_ narcosis system (Butina-Ydervan - DK 4300, Holbaek, Denmark), in a gondola with a dimension of 2.7 x 0.98 x 1.0 m (capacity of six pigs)	Head-only electrical stunner (model TL002 Gozlin) with a dimension of 65 cm x 35 cm x 18 cm
Stunning system characteristics	Head electrode: 320.43 ± 0.83 V, 0.56 ± 0.03 A. Chest electrode: 79.78 ± 0.41 V, 0.61 ± 0.08 A	CO_2_ concentration: 90.6% ±5.8	250 V and 1.3 A for 3 s

**Table 2:** Descriptive analysis of driver's sociodemographic information (age, transport training, educational status, and exclusive truck) and least square means of travel conditions (average travel speed, distance, number of stops, average stop time) during transport to three Colombian slaughterhouses.

	**Slaughterhouses**		
**Variables**	**SA**	**SB**	**SC**
**Sociodemographic**			
**Age (yr)**	35.3 (±9)^a^	39.6 (±11.2)^b^	40.5 (±7.5)^b^
**Educational status**			
Elementary school	73.7% (104)^a^	50.6% (85)^b^	9.5% (6)^c^
High school	19.8% (28)^a^	42.8% (72)^b^	90.5% (57)^c^
Professional school	5.7% (8)	5.9% (10)	0
University	0.8% (1)	0.7% (1)	0
**Transport training**			
Yes	35.5% (50)^a^	97.6% (164)^b^	4.8% (3)^c^
No	64.5% (91)^a^	2.4% (4)^b^	95.2% (60)^c^
**Farm conditions**			
**Number of farms (%, n)**	37.8% (51)^a^	55.6% (75)^b^	6.6% (9)^c^
**Presorting**			
Yes	92.9% (131)^a^	95.8% (161)^a^	98.4% (62)^b^
No	7.1%(10)^a^	4.2% (7)^a^	1.6% (1)^b^
**Load size** (average)	56.2 (±17)^a^	38(±25.7)^b^	65.3(±27.5)^c^
**Transport conditions**			
**Number of decks**			
One	7.8% (11)^a^	62.5% (105)^b^	4.8% (3)^a^
Two	86.5% (122)^a^	36.3% (61)^b^	95.2% (60)^a^
Three	5.7% (8)^a^	0.2% (2)^b^	0% (0)^b^
**Water supply**			
Yes	75.9% (34)^a^	4.8% (8)^b^	90.5% (57)^c^
No	24.1% (107)^a^	95.2% (160)^b^	9.5% (6)^c^
**Specialized transport** (yes)	100%(141)^a^	14.26%(*n =* 29)^b^	85.71%(54)^c^
**Average speed** (km/h)	60.3 (± 9.8)^a^	59.9 (± 9.2)^a^	52.1 (± 11.2)^b^
**Transport time** (h)	2.78 (±1.3)^a^	3.49 (±2.4)^b^	2.17 (±0.5)^a^
**Distance** (km)	73.1 (±46.9)^a^	92.5 (±65.6)^b^	59.9 (±20.1)^a^
**Stopovers** (yes)	1.5 (±1.2)^a^	1.5 (±0.6)^a^	2.1 (±0.8)^b^
**Average stop time** (min)	14.1 (±3.6)	14.1 (±3.6)	12.9 (±2.9)
**Space allowance** (m^2^/ 100 kg)	0.57 (±0.08)^a^	0.47 (±0.1)^b^	0.48 (±0.04)^b^

a, b, c *Different lower-case superscripts in the same row indicate differences statistically significant (p < 0.05). Slaughterhouse SA (n = 141), Slaughterhouse SB (n = 168), Slaughterhouse SC (n = 63)*.

### Demographic Information About the Livestock Drivers

This investigation included 372 livestock drivers who transported fattening pigs to the three slaughterhouses being evaluated. Truckers were interviewed to know about their age, educational status (elementary, high school, community college and university) and transport training (y/n).

### Farm and Transport Conditions

**Figure 1:**
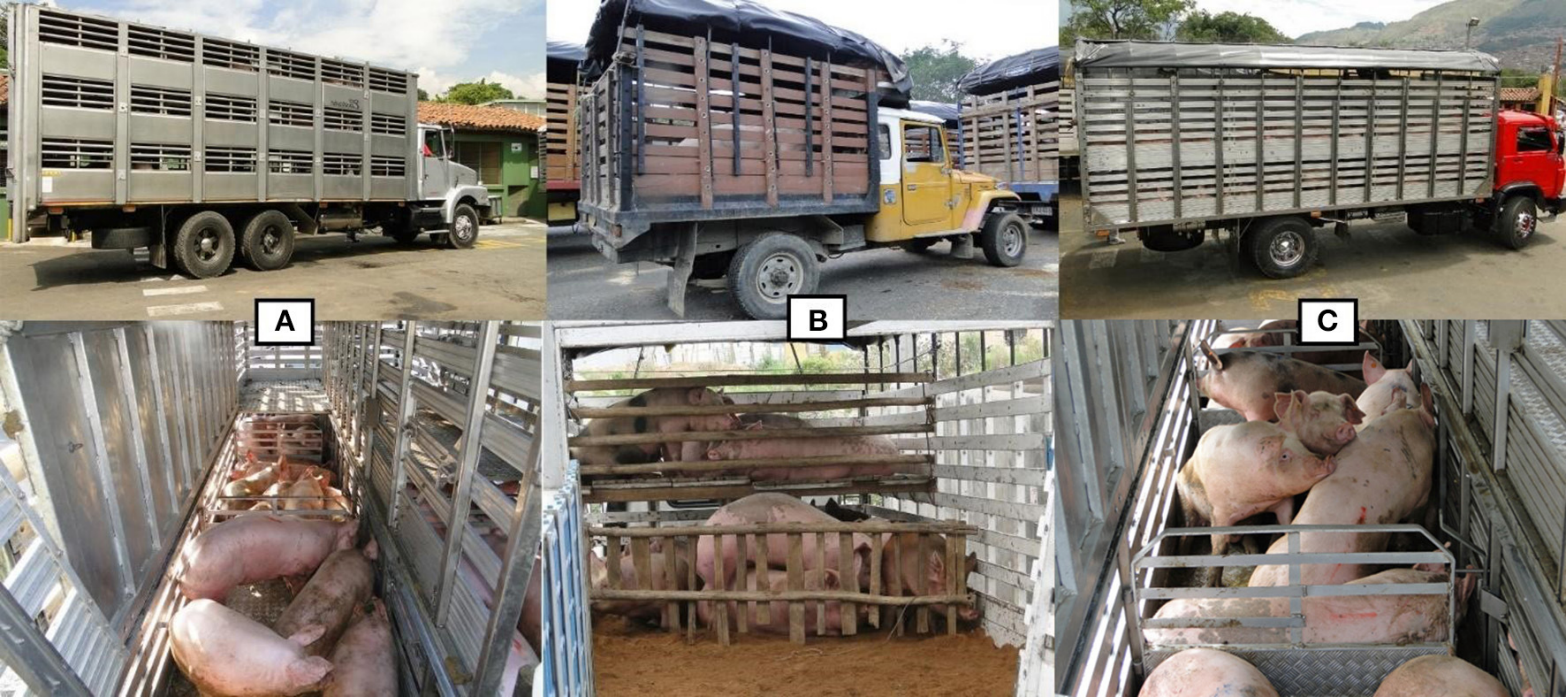
Types of trucks surveyed in this study: **(A)** Specialized triple-deck truck (Slaughterhouse **A**), **(B)** single-deck and non-specialized truck (slaughterhouse **B**) and **(C)** specialized double-deck truck (slaughterhouse **C**).

During unloading at the slaughterhouse, the following truck features were evaluated through direct observation by a trained veterinarian: number of decks (single, double, triple), water supplied (y/n), bedding material (rice husk, wood shavings), presence of roof (y/n), type of ventilation system (active, passive), type of bodywork (wood, metal, mixed), internal partitions (y/n) and space allowance (m^2^/100 kg) (see Figure 1 for the types of trucks used in the three slaughterhouses). Finally, truck drivers were interviewed to determine the following characteristics in accordance with the sanitary requirements (19): a) farm name, geographic location (department, municipality), presorting (y/n), batch size (n); b) truck model (year of fabrication), average speed (km/h), transport time (h), distance (km), stopovers (y/n), average stop time (min), specialized transport (y/n), presorting (y/n), type of roads and revisions to check for NANI and NAI pigs during transport (y/n), space allowance (m^2^/100 kg), number of dead-on arrival (DOA, n), number of NANI and NAI pigs (at arrival, unloading, lairage) and number of euthanized pigs (n).

### Identification of Non-ambulatory and Dead Pigs

Non-ambulatory pigs were defined as being unable to remain with their congeners and unable to move freely due to muscle injury or bone fracture at some stage of the transport, unloading, or lairage process (20–22). NANI were defined as pigs without obvious injury, trauma or disease, which exhibited physical signs of stress (dyspnea, red patches or discoloration of the skin, characteristic high-pitched vocalization and muscle tremors) (22). In accordance with Colombian sanitary regulations (23), NAI were subjected to emergency slaughter in the sanitary slaughter room. NANI were transferred to the observation pen and a second ante-mortem inspection was performed by a trained veterinarian. Pigs with signs of recovery were slaughtered. On-arrival, dead pigs (DOAs) were recorded as such and subjected to total condemnation (23). Additionally, the presence or absence of skin discoloration, panting, muscle tremors and vocalization were recorded during lairage. A skin discoloration was considered present when a segment of pig's skin presented a change in color in any body part without considering the tail, panting was defined as respirations carried out with the mouth open or in short gasps respirations, muscle tremors were defined as the slow and irregular vibration of any body part, or the body as a whole and lastly vocalizations were considered present if the pig displayed high pitched vocalizations (squeal/scream) during movement though the alley or chute or during lairage (24).

### Physiological Welfare Indicators

Physiological parameters measured during lairage in the pen were respiratory rate, by means of counting the movements per minute (mov/min) of the pig flank during 15 s and adjusting these values for 60 s to obtain the respiratory rate in a standard unit (respiration/min) and the rectal temperature, using a digital clinical thermometer (GLA Agricultural Electronics, San Luis Obispo, CA) inserted into the rectum of the animals for 1 min. Blood samples from non-ambulatory (NAI and NANI) pigs were collected immediately after pig's stunning into two 10 ml tubes (with and without anticoagulant). Samples were kept on ice during sampling (up to 2 h) and then taken to the laboratory for routine hematological measurements the samples were centrifuged at 2,500 rpm for 10 min (room temperature) and the serum was separated into 5 ml sterile vials and stored at 0 °C for later analyses. For the analysis of hematocrit value, EDTA was placed into the tubes as anticoagulant (2 mg/ml of blood), while the tubes without EDTA were used for blood biochemical components analysis such as glucose, urea, total protein, creatinine, unesterified fatty acids, lactate, β-hydroxybutyrate and cortisol, Packed cell volume (PCV) values were obtained using the micro hematocrit technique (25). Serum cortisol concentrations (μg/dl) were measured in duplicate using a radioimmunoassay -RIA- (Clinical Assays GammaCoat Cortisol 125I RIA Kit, DiaSorin, MN). The concentrations of glucose (mmol/l), urea (mmol/L), total protein (g/L), and creatinine (mg/dl), were determined using a Biosystem kit (Biosystems®, Barcelona, Spain), and spectrophotometer BTS-330 (Biosystems®, Barcelona, Spain). The evaluation of the unesterified fatty acids (NEFA, mmol/l) levels, β-hydroxybutyrate (mmol/L) and lactate (mmol/l) concentration were performed with Randox kits (Randox Laboratories Limited®, Crumlin, UK), and spectrophotometer BTS-330 (Biosystems®, Barcelona, Spain). The intra assay coefficient of variations CVs were 9.3, 3.9, 8.9, 4.4, 8.2, 12.7, 9.2 and 12.3% for cortisol, glucose, albumin, urea, creatinine, lactate, NEFA and β-hydroxybutyrate, respectively.

### Data Handling and Statistical Analyses

Data analyses were conducted using STATA software, version 13.0 (College Station, Texas, EU). In this study the experimental unit was a batch and an animal. A batch was defined as the group of animals transported in the same truck and unloaded at the slaughterhouse. Firstly, a normality test of the evaluated variables was carried out and the variables with non-normal distribution were transformed by means of the natural logarithm and these values were used for later statistical analysis; the results were transformed back to the original units of measures. The experimental unit was the truck load to the three slaughterhouses and a non-ambulatory pig. A total of 372 batches and 18,437 animals were included in the analysis of the risk factors. A total of 194 non-ambulatory pigs were included in the analysis of physiological variables (NANI = 145 and NAI = 49). Categorical variables (level of education, transport training, truck model, number of decks, specialized transport design, water supply, stopovers, presorting, type of bed, type of bodywork, presence of roof, presence of internal partitions, presence of non-ambulatory pigs and ante-mortem decision) were subjected to descriptive statistical analysis and presented as proportions of answers, means, and when applicable the variability was expressed as ranges. The least square means of the physiological welfare indicators the non-ambulatory pigs (dependent variables) were compared by one-way ANOVA analysis according to the slaughterhouse (independent variable) and Tukey's test to determine if there were significant differences among the three slaughterhouses evaluated (independent variable A multilevel mixed effects linear regression model analysis was performed to identify variables associated with blood concentrations of physiological indicators of stress (dependent variables) with the sociodemographic characteristics of the truck drivers, transport conditions and physical signs of stress (dyspnea, red patches or discoloration of the skin, characteristic high-pitched vocalization and muscle tremors, controlling for the effect of the farm of origin (fixed effect). Considering that the trucks present a loading capacity of 6 to 216 pigs, this variable was transformed into its logarithmic value for the analysis in order to obtain the RTTL (rate of total transport losses) (26) according to this formula:


$$\begin{array}{l} RTTL=\frac{\#\;non-ambulatory\;+dead\;pigs\;per\;truck\;}{Log\;load\;size\;}*100 \end{array}$$

For the identification of risk factors associated with the presence of total transport losses (RTTL), a multilevel mixed effects linear regression model analysis was performed, where the dependent variable was RTTL. The independent variables used were the sociodemographic characteristics of the truck drivers and the transport conditions. A backward stepwise procedure was performed to remove variables that did not account for significant variation. The model included the farm of origin and the load size as a fixed effect and covariate. The confounding effect was considered present when the estimates changed by at least 20%. The number of decks and the slaughterhouse were evaluated as confounding variables but were removed from the model because the principal variation source was the farm effect. A probability level of *P* < 0.05 was chosen as the limit for statistical significance in all tests.

## Results

### Demographic Information About the Livestock Drivers

The surveyed truckers were of heterogeneous ages, ranging from 20 to 65 years old, with truckers transporting to SA being younger than those hauling to SB and SC (*P* < 0.05) (Table 2). In the SA slaughterhouse (73.7%) a high proportion of trucker drivers had received an elementary school education, while the highest proportion of SC truck drivers (90.5%) had received a high school education. A small proportion of truck drivers had received professional school or university education (*P* < 0.05) (Table 2).

### Farm and Transport Conditions

The load size (number of pigs transported by the trucks) ranged from 6 to 120 pigs, only one truck transported 216 animals. With most single- and double-decked trucks (95.8% and 96.9%) transporting between 6 and 40 pigs and 41–80 pigs, respectively and 100% of the triple-deck trucks transported batches that ranged between 81 and 216 animals (*P* < 0.05).

The transport of fattening pigs was done in specialized trucks dedicated exclusively to this activity in a higher (*P* < 0.05) proportion in the SA and SC plants and mainly using two-deck trucks; in the SB plant, single-deck trucks were used, equipped with passive ventilation (SA and SC); only 2.4% (*n* = 4) of the trucks in the SB plant had an active ventilation system (Table 2). The year of fabrication of trucks monitored in this survey varied from 1953 to 2015, with overall most truck models being produced between 2000 and 2015. Regarding the number of truck decks, 70.6% (*n* = 84) of the single-deck trucks and 77% (*n* = 187) of the two-deck trucks were 2001–2015 models, while 80% (*n* = 8) of the triple-deck trucks were 1991–2000 models. Significant differences were observed in the provision of water for the animals during transport in the three plants evaluated (*P* < 0.05). The trucks were equipped with waterers in greater proportion in the SA (75.9%) and SC (90.5%) plants, while in SB, trucks without waterers predominated (95.2%) (Table 2).

Trucks with metal bodies predominated in SA and SC (85.1 and 77.7%, respectively); while in the SB slaughterhouse, mixed bodywork (wood and metal, 69.1%) predominated; they were equipped with a roof (89.4, 97.6, and 100%) and a small percentage with a canopy (10.6, 2.4%) and internal partitions (97.9, 73.8, and 90.4%). In the SA and SC slaughterhouses the majority of the trucks did not use bedding on the floors (92.2, 95.2%), while in SB 73.2% of the trucks were provided with rice husk and wood shavings. Travel conditions, such as transport time, distance, speed and space allowance showed significant differences (*P* < 0.05) among slaughterhouses. In all trucks there was a mixture of pigs from different batches (Table 2).

### Characteristics of Non-ambulatory and Dead Pigs

Mortality rates ranged from 0.08 to 0.17%; while prevalence rates of non-ambulatory pigs per batch ranged from 0.84 to 1.37%. In SA (76.1%) and SB (53.1%) most non-ambulatory pigs were observed on arrival during unloading; while at SC they were observed during lairage (92%) 12 h after arrival according to sanitary requirements by Colombian legislation (23) (Table 3). All pigs were not mixed and had 1 m^2^ of space available during lairage, also they had access to water *ad libitum*. 74.7% (*n* = 145) of the non-ambulatory pigs were classified as NANI and the remaining 25.3% (*n* = 49) as NAI (Ratio = 1:3). Emergency slaughter of 97% of the non-ambulatory pigs was performed in SA, while pigs with this condition were transferred to the observation pens for recovery in SB and SC. The multilevel mixed effects linear regression model identified the following as risk factors during transport associated with the total transport losses: driver (*β*: 0.002, *p* < 0.01), load size (*β*:−0.02, *p* < 0.01), speed (*β*:−0.01, *p* = 0.01), transport time (*β*: 0.1, *p* < 0.01), and the distance (*β*:−0.003, *p* < 0.01). The risk of the presence of the total transport losses did not increase according to the slaughterhouse, number of decks and the year of fabrication of trucks (Table 4).

**Table 3:** Proportions of dead-on-arrival (DOA) and non-ambulatory pigs (NA) during transport to three Colombian slaughterhouses.

	**Slaughterhouses**		
**Variables**	**SA**	**SB**	**SC**
**Pigs' Condition**			
**DOA (%**, ***n*****)**	0.08% (7)	0.15% (9)	0.17% (8)
No	92.9% (131)^a^	97.7% (164)^b^	87.3% (55)^c^
Yes	7.1% (10)^a^	2.3% (4)^b^	12.7% (8)^c^
**NANI (%**, ***n*****)**	0.6% (47)^a^	0.8% (45)^b^	1.2% (53)^c^
**NAI (%**, ***n*****)**	0.3% (20)	0.3% (19)	0.2 % (10)
**NA (%**, ***n*****)**	0.8% (67)^a^	1.0% (64)^b^	1,4% (63)^c^
**Ratio NAI:NANI**	1:4	1:2	1:5
Arrival	76.1% (51)^a^	53.1% (34)^b^	8% (5)^c^
Unloading	1.5% (1)^a^	43.8% (28)b	0
Lairage	22.4% (15)^a^	3.12% (2)^b^	92% (58)^c^
**Ante-mortem decision**			
Emergency slaughter	97% (65)^a^	12.5% (8)^b^	15.9% (10)^b^
Slaughter after rest	3 % (2)^a^	87.5 % (56)^b^	84.1% (53)^b^

a, b, c Different lower-case superscripts in the same row indicate differences statistically significant (p < 0.05). Slaughterhouse SA (n = 141, 7,933 pigs), Slaughterhouse SB (n = 168, 5,910 pigs), Slaughterhouse SC (n = 63, 4,594 pigs). ^*^It was obtained considering the total pigs in each slaughterhouse.

**Table 4:** Relationship between total transport losses (DOA + NA) per trip and transport variables^*^.

**Variables**	**Total transport losses per trip**		
	***β***	**Standard error**	***P***
**Driver**	0.002	0.00	<0.01
**Load size (** ***n*** **)**	−0.02	0.00	<0.01
**Truck speed (km/h)**	−0.01	0.00	0.01
**Transport time (h)**	0.10	0.04	<0.01
**Distance (km)**	−0.003	0.00	<0.01

* *The relationship was measured through a multilevel mixed effects linear regression model*.

Regarding the non-ambulatory pigs, 26.8% (*n* = 52) showed skin tears, 69% (*n* = 134) were panting, 55.2% (*n* = 107) showed muscle tremor and 53.1% (*n* = 103) were vocalizing. In postmortem inspection in SA, 62.7% of the pigs classified as non-ambulatory (*n* = 42) were condemned due to congestion and the presence of polyserositis (simultaneous inflammation of several serosae), 22.4% (*n* = 15) due to dermatitis problems, 16. 4% due to pericarditis heart conditions (*n* = 11), 8.9% (*n* = 6) for leg and hind leg fractures, and 7.4% (*n* = 5) for muscle injuries due to the presence of hematomas, among others.

### Physiological Welfare Indicators

The means of the physiological welfare indicators concerning animal welfare of non-ambulatory pigs in the three slaughterhouses are presented in Table 5. All physiological variables of NANI and NAI pigs were above the reference values considered normal for the swine species (27, 28), except for creatinine and β-hydroxybutyrate. Results from the multilevel mixed effects lineal regression model for transformed blood values of hematocrit, creatinine, urea, albumin and β-hydroxybutyrate showed significant (*P* < 0.05) relationships among distance and the presence of panting. Table 6 presents only the statistically significant variables associated with the physiological variables evaluated in this study.

**Table 5:** Physiological welfare indicators in blood collected from non-ambulatory pigs at three Colombian slaughterhouses.

**Variables**	**Slaughterhouses**			Reference values^***^
	**SA**	**SB**	**SC**	
Rectal temperature (°C)	39.7 ± 0.13	39.7 ± 0.12	39.5 ± 1.25	
Respiratory rate (rpm)^**^	46.6 ± 2.12^a^	43.5 ± 2.7^b^	29.8 ± 1.68^c^	8 – 18
Hematocrit (%)	41.2 ± 1.25^a^	48.6 ± 0.61^a^	49.4 ± 3.46^b^	38.3 – 43.63
Cortisol (μg/dL)	14.8 ± 0.58^a^	15.2 ± 1.09^a^	13.2 ± 0.36^b^	2.9 ± 0.10
Glucose (mmol/L)	5.4 ± 0.13^a^	6.9 ± 0.30^b^	5.1 ± 0.07^a^	4.7 – 8.33
Albumin (g/L)	4.4 ± 1.03^a^	4.5 ± 0.69^a^	17.1 ± 19.6^b^	3.1 – 3.55
Urea (mmol/L)	61.7 ± 36.1^a^	72.9 ± 28.3^b^	48.3 ± 14.9^c^	7.1 – 10.7
Creatinine (mmol/L)	2.1 ± 0.49^a^	2.3 ± 0.69^a^	2 ± 0.59^b^	3.1 – 3.55
Lactate (mmol/L)	9.1 ± 3.4^a^	5.9 ± 2.4^b^	6.1 ± 2.5^b^	0.5 – 5.50
βHBA (mmol/L)	0.1 ± 0.38^a^	0.3 ± 0.36^b^	0.6 ± 0.38^c^	0.4 ± 0.03
NEFA (mmol/L)	0.7 ± 0.38^a^	0.6 ± 0.3^a^	8.2 ± 4.04^c^	≤0.40

Slaughterhouse SA (n = 67 pigs), Slaughterhouse SB (n = 64 pigs), Slaughterhouse SC (n = 63 pigs). ^*^Super index letters to show comparisons among groups, βHBA, β-hydroxybutyrate; NEFA, Non-Steroid Fatty Acids. .

** *respirations per minute (rpm)*.

*** *Reference values from Kaneko et al. (27) and Edwards et al. (28)*.

**Table 6:** Relationships between physiological welfare indicators variables in blood from non-ambulatory pigs and transport distance and panting^*^.

**Variables**	**Hematocrit (%)**			**Creatinine**			**Urea**			**Albumin**			**β-hydroxybutyrate**		
	***β***	**SE**	**P**	***β***	**SE**	**P**	***β***	**SE**	**P**	***β***	**SE**	**P**	***β***	**SE**	**P**
**Distance**	0.001	0.001	0.01	0.001	0.000	0.6	0.001	0.000	0.02	0.003	0.001	<0.01	0.004	0.001	0.01
**Panting**	−0.283	0.048	<0.001	0.122	0.033	<0.001	0.081	0.034	0.019	−0.205	0.092	0.02	−0.053	0.126	0.6

* *The relationship was measured through a multilevel mixed effects linear regression model*.

## Discussion

Land transport is an essential part of animal husbandry; however, transport stress can produce several negative consequences for animals such as weight loss, mortality, physiological changes, and cause animals to become non-ambulatory among other factors (29). Our study compared transport conditions in three slaughterhouses, but it is important to highlight that transport conditions presented geographical differences in infrastructure and management. Therefore, it is worth considering that not all animals are handled with the same procedures due to the design of the slaughterhouses, the level of training and education of the handlers, and the presence of internal auditing systems, among other aspects. However, the evaluation of this stage allows the detection of operational aspects that slaughterhouses must take into account to ensure the fitness for transport.

### Demographic Information About the Livestock Drivers

Regarding the slaughterhouses being evaluated, transporters presented significant differences in average age, level of education and animal welfare training. Heterogeneity in the age and experience level of livestock drivers has been described in the Danish swine industry (30) and in cattle transporters in Colombia (31). Research conducted in Mexico found that the education and age of transporters did not affect their perception of animal welfare, suggesting that it is likely that daily experiences during transport mean that drivers are confronted with situations of animal suffering and that their response to pain is more associated with their empathy and attitude toward animals (29). Similar results were reported in cattle handlers in Colombian cattle markets (32) and on Brazilian farms (33).

Globally, institutions like the OIE consider that not only farmers, but also livestock drivers and transport companies are considered to be legally responsible for the fitness for transport of pigs (34). However, despite the fact that a proportion of those surveyed indicated that they had received training in animal welfare during transport, it is necessary to validate this information, because the training and qualifications of the personnel responsible for handling pigs was considered as a sanitary requirement in the 2020 Colombian “Manual de condiciones de bienestar animal propias de las especies équidas, porcinas, ovinas y caprinas” (Manual of animal welfare conditions pertaining to equines, swine, sheep and goats) (19), which is currently in the implementation process. From our experience we consider relevant that the training is based on the conditions of production and pre-slaughter, including the recommendations given by national and international academia, but more importantly, that is based on the joint work between producers and actors in the pork logistic chain and the state entities responsible for inspection, surveillance and control programs; thereby improving the dialogue and the collaborative search for solutions (35). However, the importance of the training of personnel responsible for the handling of animals pre-slaughter has been previously shown. A Canadian study evaluated the animal welfare conditions of 4,680 pigs; using a comprehensive audit protocol. In this study, the level of training of truck drivers influenced the behavior of pigs during loading and unloading (36). Another study highlighted the importance of having specialized personnel to identify and prevent stressors during the unloading and movement of pigs throughout the slaughterhouse facilities (37).

### Farm and Transport Conditions

This study is part of a Cross Sectional survey whose general objective is to characterize the commercial transport conditions of fattening pigs in three Colombian regions and to elaborate a protocol on good land transport practices. The high heterogeneity in the number of farms studied made it possible to obtain greater information on the most common transport conditions for commercial pre-slaughter.

Presorting on the pig farms of origin was a widespread management practice in this study. However, it was not possible to obtain information on pen size, pig handling conditions, number of animals selected and length of time to pre-sorting, among others, because the drivers did not have access to this information (especially at the SB plant), since the transport was not specialized, but rather carried out by contracted companies. The way pre-sorting is performed before loading has been considered a variable associated with the presence of DOAs and non-ambulatory pigs during transport in several studies (38), as well as in the reduction of loading time and some stress responses (open-mouth breathing and skin discoloration) on farm. However, pre-sorting before loading may not be a useful strategy to reduce dead and non-ambulatory pigs for farms with transport losses higher than estimated national averages in the USA (39). In the present study due to the lack of detailed information about on farm activities, presorting had not a significant effect on the presence of DOAs and NANI/NAI pigs nevertheless, there is the need for more field studies in Colombian commercial conditions to determine the role of presorting in preslaughter.

Considering that pigs at any stage of production are highly susceptible to heat stress, the design of transport vehicles and associated management practices should be carefully considered. The types of vehicles used for transporting fattening pigs in Colombia range from single-deck to double- or triple-deck trucks, with capacities up to 200 slaughter-weight pigs, equipped with mainly passive ventilation systems. Due to economic reasons, the use of double- and triple-deck trucks for pig transport is rapidly increasing in Colombia (40). However, only a low percentage of the models are equipped with hydraulic deck to facilitate animal loading and unloading. The loading and unloading of pigs through ramps imposes a strong physical effort for pigs (41), which is increased by handlers' negative intervention, resulting in increased physical effort for both pigs and handlers (42).

It is generally accepted that truck loading density has a strong influence on pig welfare and that the provision of adequate space is a key factor (22). According to Arndt et al. (43), a pig covers 0.48 ± 0.040 m^2^ when lying in a full lateral position, therefore, this aspect must be ensured, and an appropriate density allocated to allow all pigs to stand and lie in a natural body position. In this study, the highest proportion of trucks had space allowances consistent with national legislation (0.51 m^2^/100 kg) (44). However, some researchers found that official inspections and compliance with sanitary regulations have a limited effect on improving animal welfare at the farm level (35) and in pre-slaughter in general. Therefore, it would be advisable to carry out complementary studies to establish if the densities managed at the commercial level allow pigs to rest and if the requirements of the legislation are adequate and applicable to transport conditions in Colombia. In addition, it should be taken into account that pigs are marketed with weights over 100 kg in some parts of the country. Similar conditions are described in the commercialization of fattening pigs in Canada (45).

This study found a high degree of heterogeneity among the trucks evaluated according to the year of fabrication, the number of decks and transport capacity (evaluated as load size) among the different slaughterhouses. These variables were included in the multilevel regression analyses, because they are closely related to factors that affect mortality and the presence of non-ambulatory pigs during transport, such as the microclimate of the truck, the handling of pigs during loading and unloading, the space available to rest and adopt postures that facilitate heat loss and maintain balance, in addition to other factors (21, 46, 47). Other important characteristics in the level of microclimate parameters that affect animal welfare are: position of the compartment within the truck, the ventilation system of the vehicle, deck ceiling characteristics (height, thermal insulation, use of tarp), solar radiation, loading density (2), truck vibrations, the possibility of the pigs to stand or not during the trip (48) and the presence or not of internal ramps (42), among others. However, when evaluating the model for the identification of risk factors associated with the total transport losses, there was no evidence that the number of decks and year of manufacture of the truck were associated with the presence of DOAs and non-ambulatory pigs in this study.

In this study, the water supply during transport was prevalent in trucks transporting pigs to the SA and SC plants, in which transport was specialized. Colombian legislation requires that trucks transporting pigs to slaughterhouses be equipped with water troughs (44), in order to ensure animal welfare and meat safety. Deprivation of water and feed during transport can jeopardize the health and welfare of animals, with clinical (illness or disease state), physiological (changes in functional pathways), behavioral (activity, actions and interactions), and emotional (subjective experience) effects (49, 50). Likewise, it has been recognized that healthy animals transported under good conditions can better tolerate the challenges of transportation (7, 51).

### Factors Associated With the Presence of Dead and Non-ambulatory Pigs

The mortality rate reported in this study is within the range 0.07 to 5.2% and similar to that registered in other countries (13, 14, 52, 53) in pigs weighing more than 160 kg, but much higher than those reported in Brazil (0.08%) (14). The presence of DOAs during transport was associated in this study with the incidence of non-ambulatory pigs. Although pigs have developed physiological mechanisms to adapt to stressors, when the animal's ability to cope with these challenges is insufficient, death may occur (54). Additionally, it is known that mortality during transport is the product of a multi-causal problem involving several factors that can have an additional effect, such as farm size, herd size, handling, loading, transport conditions, vehicle design, among others (13, 14).

Regarding the rates of non-ambulatory pigs, the results are discordant with other studies. In a retrospective investigation conducted in Colombia with data from 3 years in one of the slaughterhouses with the highest processing at a national level, lower rates were reported (0.44–0.5%) (55). Likewise, the results are higher than those reported by other authors (14, 46). However, they are similar to those reported by Pilcher et al. (22) in USA (0.95%). The differences may be associated with factors related to the animals (genetics, age, weight), the design of the trucks, the conditions and logistics of the trip, the lower number of pigs transported in Colombian trucks, the human-animal interaction and the livestock marketing system, among others (13). Additionally, this study found a ratio of 1:3 between the frequency of NAI and NANI, following the same trend observed in studies carried out in the United States (13), but lower to those reported by other authors (21).

Ante-mortem inspection of non-ambulatory pigs demonstrated increased behavioral indicators of heat stress (panting), physiological stress (muscle tremor, increased respiratory rate and temperature) and fear (vocalizations). Increases in rectal temperature and respiratory rate are indirect indicators of heat stress and suggest a demand on physiological mechanisms that maintain body temperature (56). In commercial slaughterhouses, the slaughter of non-ambulatory pigs should be considered a priority, as observed in SA, as well as the use of devices to move non-ambulatory pigs including sleds, slide boards/belting or carts (11). In the SB and SC plants NANI were led to the observation pens mixed with pigs with other conditions, such as the presence of hernias and lameness. However, it is recommended that observation be conducted in separate pens to ensure rest and avoid injuries caused by other animals (40).

The farm effect (fixed effect) was included in the final model evaluating the total transport losses per truck and physiological variables analysis, because the farms were managed by individual operators who may have contributed to the total transport losses using different management procedures for the pigs with factors, such as high lean genetics, farm design, farm size, housing system, handling techniques, moving strategies, degree of mixing, climate control, feed withdrawal, hydration status, shipping procedures and facilities, among others, as described by Haley et al. (21) and Fitzgerald et al. (26). The caretaker's basic knowledge of the biology of the species they are working with and expertise in management are factors that contribute to minimizing the level of animal fear and stress on the farm. Other factors related to the caretaker are: personality because it influences the way the handler interacts with the surrounding environment and with the pigs. It has been documented that handlers who are likely to be less aggressive are associated with more productive herds and lower mortality rates (54). Whereas, when pigs are pre-handled in an aggressive manner, they do not have the ability to differentiate between handlers, irrespective of subsequent handling, and will therefore respond negatively to human contact, whether the experience is positive or negative (57).

Total transport losses (DOAs and non-ambulatory pigs) were associated with the truck driver in this study. Similar results were reported by Passafaro et al. (58) Who found that transport losses were lower when trucks were driven by owners rather than employees, because owners have a vested interest and therefore are more careful in handling and moving pigs throughout the transport process, compared to hired drivers. Other researchers have reported that the level of knowledge in pig behavior and the handling skills of drivers directly influences animal welfare (26). A study developed in Canada found that pigs from two farms were more reluctant to move depending on the driver in charge of loading (36), taking into account that it is the handler who generally defines the quality and initiates the interaction with the animals; therefore, subtle differences in human behavior can be crucial (59). Likewise, other authors suggest that drivers should be aware of the effect of climatic changes on animals and how to act to avoid heat or cold stress during transport (26, 30). Many factors have been associated with the effect of the driver on animal welfare such as: attitude and empathy toward animals (32, 60), years of experience as a truck driver (29), work pressure and interpersonal relationships training (59), coaching and driving style, among others (45). These results suggest that understanding in greater depth the differences between drivers may further explain transportation losses, and therefore be useful to the swine industry to contract, hire, or train truck driver (58).

The load size was included in the multilevel regression model and affected total transport losses, because this variable is directly related to the number of floors of the truck. It is important to keep in mind that large groups of animals are more difficult to handle compared to smaller groups, require more rigorous trip planning, more time for loading and unloading, and greater handling effort by personnel, among others (39). These factors can interfere with the quality of human-animal interaction, resulting in more injuries and stressed animals (14, 47). Studies conducted in the USA that evaluated transport losses found any effect of floor space being confounded with the effect of the number of pigs per compartment (group size), therefore they adjusted these variables according to the live weight of the animals (22). In future studies it would be important to evaluate other transport conditions related to load size such as the number of animals per compartment, location of pigs in the truck, duration of loading and unloading, floor space and/or loading density, truck design, and slaughterhouse characteristics, among other factors.

Truck speed was a factor that increased the likelihood of total transport losses during transport. Variations in speed during transport cause vibration and loss of balance (61) resulting in pigs' discomfort during transport (62). When exposed to vibration, body organs function as a heterogeneous group of mechanical systems causing displacement of internal organs, resulting in physiological and behavioral stress in animals (61, 63). In addition, higher speeds can affect the balance of the animals, increasing the physical effort to stand and increasing impacts between pigs and against the truck body (48).

A number of studies have reported the effect of truck design on DOAs and non-ambulatory pigs during transport (48, 64). A USA study reported that truck design was responsible for the 46.7% increase in the number of DOAs recorded between 1990 and 2002. Heterogeneity in the models of the trucks evaluated was included in this study as a confounding variable, since it is closely related to transport conditions (48) and to the different degrees of modernization achieved by producers and truckers. The use of fixed floors/decks and ramps (common in Colombian trucks) increase the risk of DOAs and the prevalence of non-ambulatory pigs, due to the difficulty of loading and unloading the animals, which increases the use of electric prods and the duration of these two procedures (12). These conditions resulted in the higher proportion of non-ambulatory pigs during unloading at SB and higher cortisol concentrations during bleeding at SC (2).

The SA and SC slaughterhouses were located in major pig production areas in Colombia, which explains the shorter transport (<4 h and <100 km) compared to the pigs slaughtered at the SB slaughterhouse (≥7 h). However, a higher incidence of non-ambulatory pigs was recorded for the shorter trips. These same results have been described in Canada (21). It has been proposed that pigs that are injured or fatigued during loading or during the first hours of travel do not have enough time to recover, because they require at least 2 h for their physiological parameters to return to basal levels (62). Other studies showed that it takes up to 2 h for pigs to accommodate and lie down in the truck (65) which would explain why pigs exhibit greater signs of stress in short trips (<1 h), compared with longer transport times and distances (22). Longer trips would allow the animals to adapt to the transport conditions and provide a rest similar to that achieved during the stay at the slaughterhouse, if transport conditions are optimal (e.g., loading density) (13). However, the additive effects of long-term feed and water deprivation, vibrations, loss of balance, heat stress and other factors on animal fatigue must be considered.

### Physiological Welfare Indicators

During transport and lairage, pigs must make physiological adaptations to respond to periods without access to feed and water. Dyspnea is the most frequently observed sign of stress in NANI in response to aggressive handling, distance traveled during handling and loading and unloading conditions (20), which may be also related to the farm of origin, i.e., poor microclimate control resulting in greater ammonia level in the air and post-mortem sign of pneumonia (36). During this study in the SA, seizures due to lung lesions were frequent in non-ambulatory pigs. Pigs with lung lesions present hyperventilation to maintain blood pH at a level close to homeostasis and are more susceptible to develop respiratory acidosis due to their limited ability to eliminate carbon dioxide from their system, which is accentuated when the animals are subjected to a stressful event such as transport and handling (66). On the other hand, vocalizations were frequent in NANI, as a possible indicator of stress and fear. In addition, vocalizations could play an important role in the transfer of emotions between individuals by means of “emotional contagion” or “empathy”, which could result in an impairment of animal welfare at the group level based on stress in an individual group member (67). This is an aspect that is not well documented and is an opportunity for further research to understand how emotional states of animals can affect their social behavior.

This study suggests that a large proportion of non-ambulatory pigs did not recover during lairage as shown by the serum cortisol and glucose values that were above the baseline values (27, 68). The increase in glycaemia in non-ambulatory pigs can be attributed to the energy demand caused by the difficulty of handling and transport (53). On the other hand, the higher lactate could have been a response to acute exercise and an increase in oxygen demand, which activated the anaerobic glycolytic pathway, which produces metabolic acidosis, characterized by a decrease in blood pH and an increase in body temperature between 1 and 2.5°C, known as stress hyperthermia (64), an aspect that was observed in this study and is a criterion considered as an indicator of stress and well-being (69). Likewise, as an indicator of dehydration, all the pigs in the study had increased hematocrit and blood albumin concentrations.

The distance traveled was strongly and significantly related to the blood concentrations of some physiological welfare indicators (hematocrit, creatinine, urea, albumin and β-hydroxybutyrate) of NANI and NAI pigs, taking into account the effect of the animals' farm of origin. However, in this study, farm visits were not conducted and therefore, relevant information related to animal handling, preparation for travel, fasting conditions, loading, managerial and administrative aspects, personnel training, as well as other factors that may have a stressor effect on the animals were not considered. Understanding the factors associated with the presence of DOAs and non-ambulatory pigs (total transport losses) can be of help in decision making and the development of transport strategies to minimize risk and decrease economic losses during swine pre-slaughter, as described by Passafaro et al. (58). It is important to remember that previous research has shown that multiple concurrent interacting factors such as handling intensity, transport floor space, water and food deprivation and distance moved during handling, among others, have additional effects on the metabolic responses of non-ambulatory pigs (NANI, NAI and DOAs) (20, 58). Based on these findings, it is essential to promote the implementation of good transport practices under commercial conditions and the training of personnel responsible for handling the animals to minimize the negative effects of pre-slaughter on animal welfare and the associated economic losses.

The NANI/NAI pigs are a problem to the industry due to the economic losses that represent for producers and the resultant decreased meat quality and condemnations (14), but there is a big ethical issue that surrounds them that needs more attention. These pigs suffer due to their conditions of arrival at the slaughterhouse and the economical threshold determined for acceptance is sometimes not enough to avoid their presence at slaughterhouses, this is a major concern for consumers who are being more invested in the source of meat they are eating (70–72) and activist organizations or individuals (73) who find this is a major concern that impact their decisions and perspective over the animal industry. Although researchers, consumer and producers are all involved in an effort to diminish animal suffering (35), this present scenario where the supply chain is segmented and responsibility besides economic losses is nonexistent (74) becomes a setback in the impact of improving activities. It's necessary then, to have an ethical scope when evaluating and designing strategies that help diminish the prevalence of NANI/NAI pigs to not just a place where economic losses are minimal but where is ethically accepted.

Two limitations have been identified in this study (1) selection biases, due to the difficulty in generalizing the results in different geographic areas and the selection and evaluation of the farms, and (2) measurement biases, because the Cross-Sectional study design did not allow for tracking the animals from the farm to the slaughterhouse. The first bias was controlled by the authors through a multilevel analysis by generalized linear mixed models to control the effect of the farm of origin and the control of confusion variables such as the number of decks on the truck and the slaughterhouse. The second bias was partially controlled by multivariate statistical analyses and the training of the veterinarian responsible for the evaluation of the trucks and the application of the structured instrument. Future research needs to measure on-farm and other environmental variables during transport to identify additional stress factors to guide transport recommendations under commercial conditions.

## Conclusions

In this field study involving three commercial slaughterhouses in Colombia, truck speed, transport time, truck driver and distance traveled were identified as risk factors that increased the probability of total transport losses (DOAs and non-ambulatory pigs) during land transport. Other factors, such as load size and differences between the evaluated farms of origin were associated with these indicators. The analysis of physiological indicators suggests that NANI pigs did not recover during lairage, thus necessitating the need for a more accurate analysis of the physiological indicators, prioritizing the ante-mortem inspection of pigs to perform emergency slaughter. More research in commercial conditions that involve drivers is needed to develop effective strategies to improve Colombian pig's transportation chain.

## Data Availability Statement

The original contributions presented in the study are included in the article/supplementary material, further inquiries can be directed to the corresponding author.

## Ethics Statement

The animal study was reviewed and approved by the Ethics Committee for Animal Experimentation of the University of Caldas (Act 1 14/07/2015, -Activities with minimal risk-).

## Author Contributions

MR assisted with the conception and design of the experiment, preparation, data analysis, and preparation of the figures and manuscript. RH prepared the data for analysis and analyzed the data under the guidance of MR and JS. MR, JS, and RH contributed to interpretation of the results. MR and JS drafted and edited the manuscript. All authors read and approved the final manuscript.

## Funding

This work was based on research supported in part by Research Investigation and vice chancellor of the University of Caldas and the Doctorate in Agrarian Sciences and Master in Veterinary science programs of the University of Caldas.

## Conflict of Interest

The authors declare that the research was conducted in the absence of any commercial or financial relationships that could be construed as a potential conflict of interest.

## Publisher's Note

All claims expressed in this article are solely those of the authors and do not necessarily represent those of their affiliated organizations, or those of the publisher, the editors and the reviewers. Any product that may be evaluated in this article, or claim that may be made by its manufacturer, is not guaranteed or endorsed by the publisher.
